# Longitudinal Links of Individual and Collective Morality with Adolescents’ Peer Aggression

**DOI:** 10.1007/s10964-021-01518-9

**Published:** 2021-10-18

**Authors:** Gianluca Gini, Robert Thornberg, Kay Bussey, Federica Angelini, Tiziana Pozzoli

**Affiliations:** 1 Department of Developmental Psychology and Socialization, University of Padua, Padova, Italy; 2 Department of Behavioural Sciences and Learning, Linköping University, Linköping, Sweden; 3 Department of Psychology, Macquarie University, Sydney, Australia

**Keywords:** Reactive aggression:, Proactive aggression;, Moral identity, Moral disengagement, Class norms

## Abstract

Adolescents’ aggressive behavior has been often linked to biases in morality. However, limited knowledge is available regarding the relative strength of different moral correlates, both at the individual and class-level, in predicting different types of aggressive behavior over time. To address this gap, the present study tested the prospective associations of moral identity and moral disengagement with reactive and proactive aggression in a short-term longitudinal study. The sample consisted of 1158 Italian adolescents (48.7% females; *M*_age_ = 13.6 years, SD = 1.1). Participants completed self-report measures of moral identity, moral disengagement, perceived collective moral disengagement in the fall, and reactive and proactive aggression in the fall and in the spring. Multivariate multilevel analysis indicated that, at the individual level, after controlling for the stability of aggressive behavior, T2 (Time 2) reactive aggression was higher for students who reported lower moral identity and higher moral disengagement at T1 (Time 1). For proactive aggression, a significant interaction effect indicated that the negative association between T1 moral identity and T2 aggression was apparent only at high levels of T1 moral disengagement. Moreover, proactive aggression was significantly predicted by higher perceived collective moral disengagement. At the class-level, T1 collective moral disengagement helped explain between-class variability of T2 reactive and proactive aggressive behavior. How these results expand previous research on morality and aggressive behavior and their potential implications for prevention and intervention programs is discussed.

## Introduction

Risk factors for the development of aggressive behavior toward peers have been studied for decades (Lansford, 2018). Personal correlates of youth’s aggressive behavior include multiple factors in the cognitive, emotional, motivational, and moral domains. Related to the latter, aggressive behavior is inherently “immoral”, because it causes harm to the victim; it is therefore not surprising that interest in the moral correlates of peer aggression has increased during the last two decades. However, research gaps still exist, especially with a lack of integration of different moral correlates into a single model that allows testing the relative strength of each moral dimension as well as their interactive effects. Another important gap in this literature relates to the very limited analysis of class-level moral dimensions, especially in a longitudinal design. To begin to fill these gaps, the present study combined two moral constructs that have been rarely analyzed together in the aggression literature involving adolescents, namely moral identity and moral disengagement. Adopting a longitudinal, multilevel approach, this study aimed to test the prospective associations of adolescents’ moral identity and moral disengagement, the latter measured both as an individual trait-like characteristic and as a class-level, collective dimension, with adolescents’ aggressive behavior over six months. Finally, another noteworthy research gap is that previous studies have usually adopted general measures of aggressive behavior, without distinguishing different types based on the underlying motivation (i.e., whether the aggression is reactive or proactive), or focused only on particular categories of aggressive behavior such as school bullying. Therefore, to further expand the current research on youth’s morality and aggression, the present study included both reactive and proactive aggression as outcomes.

### Reactive and Proactive Aggression

Considering the underlying purposes that aggression may serve, two functions of aggressive behavior can be distinguished, namely reactive and proactive aggression (Little et al., 2003). Briefly, reactive aggression is defensive or retaliatory, and occurs in response to real or perceived provocation. Conversely, proactive aggression is unprovoked and is used to attain a goal, such as social dominance and status (Hubbard et al., 2010). Of course, there is a significant overlap between the two types of aggression, but they are also conceptually distinguishable and can have somewhat different correlates (e.g., Polman et al., 2007). Quite surprisingly, little research has explicitly focused on moral aspects of the reactive versus proactive distinction (e.g., Arsenio & Gold, 2006), and even less in adolescence. Disentangling the role of different moral components in the two forms of aggression could contribute to the theories about the distinction between reactive and proactive aggression (e.g., Arsenio et al., 2009), on one hand, and could have important implications for more targeted intervention efforts, on the other hand. Hence, the present study aimed to explore whether the combination of the moral dimensions under study had similar or different longitudinal associations with adolescents’ reactive and proactive aggression. We focused specifically on adolescence because in this developmental period most youth have internalized a suite of moral rules (Marshall & Marshall, 2018) and developed a moral identity (Krettenauer & Victor, 2017); moreover, the association between moral disengagement and aggressive behavior is stronger among adolescents than among children (Gini, Pozzoli, & Hymel, 2014).

### Moral Identity and Aggressive Behavior

In this study, morality is defined as conceptions of human welfare, justice and rights, and regulation of actions that affect others in these terms (Nucci, 2001). While children’s moral reasoning tends to be based on the need for approval, during adolescence adherence to moral decisions that prescribe what is right and wrong becomes progressively more internalized (Gibbs, 2003). During development, understanding of moral elements also changes, so that children tend to emphasize visible, physical harm, while adolescents become able to understand more abstract issues of unfairness, including equitable treatment of all people (Nucci, 2001). Adolescents’ moral development also includes the understanding of what impacts human welfare and principles of justice (Turiel, 2002).

Specifically, moral identity refers to a person’s perception of how important moral qualities are to their self-concept or, stated in other terms, the degree to which being a moral person is important to an individual’s identity (Hardy et al., 2020). Moral identity is usually conceptualized as something that is relatively stable across situations—like a personality trait or chronically accessible moral schema (Narvaez et al., 2006) that may be more or less activated in particular situations (Aquino et al., 2009). It also develops over time (Moshman, 2011) and is an important part of identity formation that occurs during adolescence. For some adolescents it is a more central aspect of their self than for others. In addition, there may be individual differences in labeling certain actions as moral (Hardy & Carlo, 2011) and, to some extent, what “being a moral person” means may vary among particular subgroups of people (Maitra et al., 2018). Nonetheless, consistent with the long tradition of research on moral development, in this study moral identity is conceptualized and measured according to the above definition of morality, thus considering individuals with high moral identity those who endorse moral values such as being honest, compassionate, fair, and generous are central for defining their personal identity (Hertz & Krettenauer, 2016).

The importance of moral identity in the more general, subjective sense of personal identity can vary among people. Some people conceive morality as essential to define who they are, whereas others consider other characteristics or competencies (e.g., in the cognitive or athletic domain) as more important than moral qualities in defining their identity (e.g., Blasi, 1993). People with a higher moral identity have stronger moral motivation to behave accordingly (i.e., prosocially) and to avoid immoral behavior (i.e., antisocial and aggressive behavior), so that there is consistency between their behaviors and self-image (Hardy et al., 2020). In other words, the link between moral identity and moral/immoral behavior may be explained in terms of the individual’s striving for self-consistency, as described in Blasi’s Self Model (1983). According to this model, morality can represent a driving force in guiding behavior for people who consider being a moral person as a central part of their self.

Research has indeed shown that high centrality of moral characteristics for defining one’s self is positively associated with prosocial behavior, whereas it is negatively associated with the enactment of aggressive and antisocial behavior (Hertz & Krettenauer, 2016). In a cross-sectional study with a sample of American adolescents, moral identity—in terms of the moral ideal self—was found to be negatively associated with aggressive behavior (Hardy et al., 2014). More recently, the role of adolescents’ moral identity has been analyzed especially in the context of school bullying. For example, in a cross-sectional study with Italian adolescents (Pozzoli et al., 2016), moral identity was negatively associated with bullying behavior, even after accounting for individual levels of moral disengagement in the same model. A similar negative association between moral identity and bullying behavior has subsequently been found in a Chinese sample (Teng et al., 2020). However, bullying is not the sole type of aggressive behavior among adolescents and research on the role of moral identity in youth’s reactive and proactive aggression is certainly warranted, especially in a longitudinal design and in conjunction with other moral dimensions. In the present study, therefore, adolescents’ moral identity was included as one key individual predictor of reactive and proactive aggression. Even though the lack of studies explicitly testing the role of moral identity in the two forms of aggressive behavior makes it difficult to formulate specific a-priori hypotheses about possible differences, there are not conceptual reasons to hypothesize that high moral identity is a protective factor for one form of aggression but not for the other one. Instead, youth who define their identity based on moral qualities should be equally less likely to engage in either form of aggressive behavior than peers with lower moral identity.

### Individual Moral Disengagement and Aggressive Behavior

Acting against one’s moral standards typically brings self-condemnation in the form of discomforting feelings such as guilt or shame (Bandura, 2002). However, according to the social cognitive theory of moral agency, people can use psychological strategies to avoid the self-sanctions that could follow immoral conduct, thus preserving one’s self-concept as a moral person and psychological wellbeing (Bandura, 2016). These strategies include framing the negative behavior in a positive light and diminishing its severity, obscuring or minimizing one’s responsibility, minimizing or distorting the consequences of one’s action, or dehumanizing or blaming the victim (Bandura, 2016).

In the last three decades, the concept of moral disengagement has been proposed as a risk factor for the enactment of a variety of youth’s negative conduct, including general aggressive behavior, bullying, and other violent behavior. Findings within this literature are quite consistent and support the notion that individuals’ tendency to morally disengage is positively associated with different types of peer aggression among children and adolescents (Killer et al., 2019). Importantly, the results of one meta-analysis showed that the association between moral disengagement and adolescents’ aggressive behavior is almost twice as strong as that of children (Gini, Pozzoli, & Hymel, 2014), indicating that adolescence is a stage of life in which this link becomes particularly important and suggesting the need to further analyze developmental changes. Unfortunately, the vast majority of studies have used a cross-sectional design, whereas the longitudinal association between moral disengagement and aggressive behavior during adolescence has been analyzed to a lesser extent. For example, in a short-term longitudinal study with Australian adolescents (Barchia & Bussey, 2011a), moral disengagement was found to predict aggression 8 months later. Moreover, moral disengagement was found to predict bullying perpetration 6 months later among American adolescents (Wang et al., 2017). Similar findings about the predictive role of moral disengagement in the context of school bullying have been reported in other studies across different countries, such as Sweden (Bjärehed et al., 2021) and Switzerland (Sticca & Perren, 2015). In a study that employed person-centered analyses, adolescents who maintained constant medium-high levels of moral disengagement over the course of 6 years reported the highest levels of physical and verbal aggressive behavior (Paciello et al., 2008). Despite the majority of available studies having confirmed a positive association between moral disengagement and bullying or general aggression over time, a few studies have failed to find a significant longitudinal association when moral disengagement was included in a more complex model with other predictors (Orue & Calvete, 2019). It is therefore important to continue to study whether and under what circumstances moral disengagement constitutes a longitudinal risk factor for aggressive behavior in adolescence.

Beyond the limited number of longitudinal studies compared to cross-sectional ones, another gap of the current literature is the lack of knowledge about the relative role of moral disengagement for reactive and proactive aggression. A meta-analysis that compared the strength of the link between moral disengagement and general aggression with that between moral disengagement and bullying (i.e., the most common form of proactive aggression among school-aged youth) reported comparable effect sizes (Gini, Pozzoli, & Hymel, 2014). However, the sample of studies included in that meta-analysis did not allow an explicit comparison between reactive and proactive aggression. Studies published in the following years have not filled this gap yet. In one of the few available studies, which employed a cross-sectional design, moral disengagement was positively and similarly associated with both reactive and proactive aggression in a sample of Italian adolescents (Gini et al., 2015a). Therefore, to date it remains unclear whether we should expect a stronger longitudinal link for one type of aggression compared to the other, or whether moral disengagement is a general mechanism that functions equally in any type of aggressive behavior. Based on the limited empirical evidence available, it is expected to find comparable effects of individual moral disengagement on both forms of aggression over a period of 6 months.

Finally, although much research has focused on the main effects of moral disengagement on youth’s aggressive behavior, less is known about how moral disengagement interacts with other individual risk factors, particularly other moral characteristics, that increase the propensity of some youth to engage in aggression. For example, a few studies have showed the moderating role of moral disengagement in the relationship between low empathy and antisocial behavior among adolescents (Bussey et al., 2015), or between psychopathic traits and reactive and proactive aggression (Gini et al., 2015a). The only study that has tested the interaction between moral disengagement and moral identity among youth has found that aggression was higher for adolescents who had lower levels of moral identity and higher levels of moral disengagement (Hardy et al., 2015). This finding indicates that not only both low moral identity and high moral disengagement increase the risk for aggressive behavior, but that their co-occurrence can have a negative synergistic effect. Because this effect was found in a cross-sectional study, this study aimed to replicate and expand this finding testing whether moral disengagement interacted with moral identity to longitudinally predict adolescents’ aggressive behavior. The lack of integrated models that test the interplay of moral disengagement with other moral dimensions, such as moral identity, is a clear gap in the literature that this study aims to fill.

### Collective Moral Disengagement and Aggressive Behavior

Even though moral disengagement has been most frequently studied at the individual level, according to social cognitive theory (Bandura, 2016) moral behavior is determined by a combination of personal and social influences. Moral agency is cultivated and learned within the community in which individuals develop significant social relationships. Group processes can indeed facilitate (or inhibit) immoral behavior by virtue of the responsibility being shifted to the collective as opposed to the individual: for example, people are more likely to behave more cruelly in a group as opposed to when they are alone; likewise, group members can sometimes share a negative perception of the victim or blame the victim for his/her condition (Haslam, 2006). Specifically, referring to moral disengagement processes at the group-level, *collective moral disengagement* has been defined as “an emergent group-level property arising from the interactive, coordinative, and synergistic group dynamics” (White et al., 2009, p.43). This concept is embedded within the conceptual framework of social cognitive theory that includes not only personal agency but also collective agency as a key feature of the self-regulatory process (Bandura, 2002). As for other collective constructs, such as collective efficacy (Barchia & Bussey, 2011b), collective moral disengagement operates through similar processes to individual moral disengagement, differing only in the unit of agency. That is, collective moral disengagement includes the same mechanisms as individual moral disengagement, but it refers to the beliefs in justifying negative actions that are—to some extent—shared within a significant social group.

The construct of collective moral disengagement can be considered from both an individual and a group perspective (Gini, Pozzoli, & Bussey, 2014). At the individual level, it refers to individuals’ beliefs about the extent to which members of their group use moral disengagement mechanisms in everyday interactions; in other words, it refers to individuals’ perception of the degree to which morally disengaged justifications are shared by members of their group. Following previous studies, in this work this concept was labeled “perceived collective moral disengagement” (Gini et al., 2015b). Beyond the individual tendency to morally disengage, believing that peers within a given group justify aggressive behavior, view it as “normal” and an acceptable way of interacting with others without suffering negative consequences, or blame the victim for their suffering, may increase the likelihood of aggressing against others. At the group level, collective moral disengagement refers to the collective property of a given group, that is, it does not reflect individual members’ use of moral disengagement mechanisms but the degree to which such mechanisms are shared by members within that group.

Collective moral disengagement in adolescence is especially important as the peer group and its norms assume prominence during this stage of development (Brechwald & Prinstein, 2011); however, much less is known about collective moral disengagement compared to individual moral disengagement and several research gaps still exist. The first gap consists in a limited knowledge of the role of collective moral disengagement in aggression among classmates. Indeed, to date, only a few studies have empirically analyzed this link. In a first study, it has been found that both individual moral disengagement and student perceived collective moral disengagement (measured at the individual level) were associated with aggressive behavior in a sample of Italian adolescents (Gini et al., 2015b). Moreover, at the class-level, it was found that peer aggression was more likely in school classes characterized by higher levels of collective moral disengagement. These findings have been subsequently confirmed in a few studies conducted in other countries, such as Sweden (Bjärehed et al., 2019; Thornberg et al., 2021) and Czech Republic (Kollerová et al., 2018). However, almost all the existing empirical evidence is based on cross-sectional data. Only one study has longitudinally tested the association between collective moral disengagement and youth’s aggressive behavior (specifically bullying) reporting somewhat mixed results (Thornberg et al., 2019). Although changes in classroom collective moral disengagement were not found to be associated with changes in bullying over one school year, a significant longitudinal correlation between classroom collective moral disengagement and bullying at the classroom level did emerge. While preliminary evidence exists suggesting that high collective moral disengagement be considered a concurrent risk factor for class-level aggressive behavior, more research efforts that test this link longitudinally are required. Moreover, previous studies have tested the role of collective moral disengagement either in the specific context of school bullying or in general peer aggression with none explicitly analyzing reactive and proactive aggression separately. The present study aimed to contribute to filling these gaps. The hypothesis was to confirm collective moral disengagement as a longitudinal risk factor for both forms of aggressive behavior. However, considering the importance of peer group processes (peer pressure, group norms, homophily, etc.) for proactive aggression (e.g., Salmivalli, 2010), it is also plausible that collective moral disengagement plays a stronger role in this form of aggression. That is, it may be hypothesized that youth who perceive that morally disengaged justifications are shared by their peers, who therefore may easily justify aggressive behavior, could report to enact more proactive aggression than their classmates after 6 months, because this behavior would be considered more acceptable and, to some extent, a way to promote their status within the group. Reactive aggression, instead, should be more motivated by personal factors, such as perceived hostility (Arsenio et al., 2009), anger or impulsivity (Hubbard et al., 2010), than by group norms.

Finally, as for individual moral disengagement, research that tests the relative contribution and interaction of perceived collective moral disengagement with the other moral dimensions is scarce. Specifically, one previous study has found that perceived collective moral disengagement moderated the association between individual moral disengagement and peer aggression (Gini et al., 2015b), indicating that this link was significant only when adolescents also perceived that moral disengagement mechanisms were shared among classmates. While this moderation effect was found in a cross-sectional study, this study aimed to replicate this pattern longitudinally. Furthermore, no studies have tested the possible interaction between perceived collective moral disengagement and moral identity. It could be hypothesized that moral identity might mitigate the positive association between collective moral disengagement and aggressive behavior, that is, youth with higher levels of moral identity may report less aggressive behavior than their classmates even if they perceive a “morally disengaged” climate among peers.

## Current Study

An important research gap that still exists is the lack of analysis of moral identity and (individual and collective) moral disengagement together in a single study able to test the relative strength of each moral dimension as well as their interactive effects. In addition, there is still a very limited analysis of class-level collective moral disengagement, especially in a longitudinal design. Finally, studies on the moral correlates of adolescents’ aggressive behavior have rarely analyzed reactive and proactive aggression together. To contribute addressing these issues, the aim of the current study was to examine whether individual-level morality (i.e., moral identity, moral disengagement, and perceived collective moral disengagement) and class-level morality (i.e., classroom collective moral disengagement) were associated with peer aggression over the course of the same school-year in a sample of adolescents. First, it was hypothesized that the three individual-level moral predictors, measured in the fall, would be significant protective or risk factors for aggressive behavior 6 months later. These expectations were based both on the tenets of theories of self and social cognitive theory of moral agency and on previous cross-sectional and longitudinal findings reviewed above. Moreover, possible moderation effects were tested by including the two-way interactions between the individual-level moral dimensions, that is, the interactions between (i) moral identity and individual moral disengagement, (ii) moral identity and perceive collective moral disengagement, and (iii) individual moral disengagement and perceive collective moral disengagement. Moreover, to further add to the existing literature both reactive and proactive aggression were included as outcomes exploring whether similar or different effects would emerge.

At the class-level, it was hypothesized a positive longitudinal association between collective moral disengagement and both forms of aggressive behavior. That is, it was expected that aggression in the spring would be more likely in school classes that reported higher collective moral disengagement in the fall, even after controlling for the class-level stability of aggressive behavior. Finally, it was examined whether there was between-class variability in the associations between individual predictors and T2 aggression, and whether any variation could be explained by collective moral disengagement (i.e., cross-level interaction).

## Methods

### Participants

The data used in this study are taken from a larger dataset of a longitudinal project about social-cognitive and moral correlates of adolescents’ aggressive behavior. Part of that dataset has been previously used in another study that focused on defending behavior in bullying (Gini, Pozzoli, Angelini, Thornberg, & Demaray, in press). There is only minimal overlap between the data used in the previous study and those used in the current study: moral identity and individual moral disengagement are the only variables used in both studies, and these variables address distinct research questions in this study.

Classrooms were sampled from 9 public schools located in urban and suburban areas in the North of Italy. In total, 67 classes (grades 7^th^–10^th^; in Italy, students typically enter grade 7^th^ when they are on average 12-year-old) were involved. The average class size was 18.3 (SD = 4.3) students. It is important to note that in the Italian school setting the classroom represents the most meaningful unit of peer clustering being a very stable social context. Students stay in a single classroom with the same group of peers every day for the whole school year, irrespective of the subject to be taught. As such, students’ relationships within a given classroom are usually more frequent and significant than those with students from other classrooms. This, together with the small number of schools included in the sample, is the reason why, following previous studies (e.g., Gini et al., 2015b), collective moral disengagement was conceptualized and measured at the class-level, and not at the school-level.

The first wave of data collection took part in December 2017, after about 3 months from the beginning of the school year. At that time, a total of 1299 students (48.3% girls, mean age = 13.6 years, *SD* = 1.1) completed the study measures. Due to students being absent from school or having changed school, 1172 students (48.7% girls) participated in the second wave after about 6 months (end of May 2018, that is, almost the end of the school year), yielding a retention rate of 90.2%. Of these, 14 students did not respond to the reactive-proactive aggression scale, therefore the final sample at T2 included in the current analyses was composed of a total of 1158 students. Attrition analyses were performed to examine group differences between students who participated in both waves and those who did not. It was found that there was no differential attrition based on sex (*χ*^2^ = 0.70, *p* = 0.71). Moreover, no significant differences emerged in any of the study predictors (Results are reported in Appendix 1 - Supplementary material). However, students who did not participate at T2 were slightly older than students who participated in both waves (*M*_age_ = 14.18 vs. *M*_age_ = 13.60; *t* = 5.44, *p* < 0.001).

In terms of ethnic/cultural background, consistent with national statistics about the Italian student population (Miur, 2019), for the majority of participants both parents were born in Italy (88.9%), and 11.1% of students had one or both parents born in foreign countries. Socioeconomic background was assessed through the Family Affluence Scale III (FAS; Torsheim et al., 2016), which is a validated proxy measure for family SES. Sample items of this measure assess how many computer devices are available at home or whether a washing machine is available. Most of the participants came from middle-class families (low FAS: 7.2%; medium FAS: 59.7%; high FAS: 33.1%).

### Procedure

Participation of classes in the study was first authorized by school principals. Then, participants’ parents provided an active consent for participation in both waves. Less than 10% of students in the participating classrooms did not receive parental consent. Before data collection, assent for participation was also obtained from each of the adolescents with parental consent. They were informed that participation in the study was voluntary and they could refuse to participate or withdraw from the study at any time. None of the participants refused to participate.

Data were collected twice in each classroom during one school year; the participants filled out a web-based questionnaire on computers in their regular classrooms. T1 and T2 data were matched with an anonymized alphanumeric code. A graduate research assistant was present during the data-collection sessions and informed the participants that their answers would be treated anonymously and that they could raise their hand if they needed assistance (e.g., to clarify items or words of the questionnaire). At the end of data collection, any questions about the content of the questionnaires or the general aims of the study were answered. The study protocol was approved by the Ethics Committee for Research in Psychology of the first author’s University (protocol #1157/2012).

### Measures

#### Reactive and proactive aggression (T1 and T2)

At both waves a 24-item scale (Little et al., 2003) was used to measure participants’ reactive and proactive aggressive behavior. Specifically, 12 items described reactive aggression (including overt and relational items; e.g., “When I’m hurt by someone, I often fight back,” “If others upset or hurt me, I often tell my friends to stop liking them”) and 12 items measured proactive aggression (including overt and relational items; e.g., “I often start fights to get what I want,” “I often tell my friends to stop liking someone to get what I want”). For each item, participants reported the frequency of their aggressive behavior using a 6-point scale (from 1 = *not at all* to 6 = *very much*).

This scale has been already used with Italian adolescents, showing good psychometric qualities (e.g., Gini et al., 2015a). Moreover, both internal and external (e.g., criterion) validity of the scale have been demonstrated (Little et al., 2003). For this study, longitudinal scalar invariance was confirmed (see the Results section and Table 1 for details). Therefore, for each participant, answers to the respective items were averaged to obtain a score for reactive aggression and one for proactive aggression at T1 (reactive aggression: Cronbach’s α = 0.89; 95% CI = 0.98–0.90, McDonald’s ω = 0.89; proactive aggression: α = 0.95; 95% CI = 0.94–0.95, ω = 0.95) and T2 (reactive aggression: α = 0.89; 95% CI = 0.88–0.90, ω = 0.89; proactive aggression: α = 0.96; 95% CI = 0.95–0.96, ω = 0.96).

**Table 1:** Fit indices for longitudinal invariance tests of aggression scores

Two-Factor Model	χ^2^	df	CFI	ΔCFI	RMSEA	ΔRMSEA	SRMR	ΔSRMR
Configural invariance	7857.4	1074	0.931		0.068		0.071	
Metric invariance	6718.1	1096	0.942	−0.011	0.062	0.006	0.072	−0.001
Scalar invariance	6611.6	1216	0.945	−0.003	0.057	0.005	0.072	0.000

Moreover, consistent with studies that analyzed class norms and other class characteristics (Busching & Krahé, 2020; Kollerová et al., 2018; Salmivalli & Voeten, 2004; Szumski et al., 2020; Yun & Graham, 2018), class-level indicators of reactive and proactive aggression were created by averaging the individual reactive aggression scores and proactive aggression scores at T1 for each classroom. These scores, labeled “class-level aggression”, were used in the multilevel model to control for the stability of the respective aggressive behavior from T1 to T2.

#### Moral identity

Participants completed an adapted version of the Good-Self Assessment (Barriga et al., 2001), previously developed and used with Italian adolescents (Pozzoli et al., 2016), measuring the centrality of moral characteristics to an individual’s self-concept. Participants were asked to rate 16 items that asked: “How important is it to you that you are…[*personal characteristic*]?” The response scale ranged from 1 (*not important to me*) to 5 (*extremely important to me*). Eight items included a moral characteristic (fair, generous, honest, kind, respectful, sincere, trustworthy, altruist) while the other 8 referred to non-moral, but equally desirable characteristics (autonomous, brave, brilliant, creative, humorous, intelligent, pleasant, sociable). The list of qualities had been pretested with adolescents for their degree of morality (which was rated significantly higher in the former category), and valence and desirability (rated as very similar for the two categories; for details about this pretest, see Pozzoli et al., 2016). The factorial distinction between moral and non-moral characteristics has been confirmed both in the original study (Barriga et al., 2001) and for the Italian adaptation of the instrument (Pozzoli et al., 2016). Moreover, convergent validity was found with this measure significantly correlating with (low) self-serving cognitive distortions (Barriga et al., 2001).

Following the procedure described by Barriga and colleagues (2001) (see also Pozzoli et al., 2016), the average score in the non-moral items (*α* = 0.74; 95% CI = 0.72–0.76, *ω* = 0.74) was subtracted from the average score in the moral items (*α* = 0.85; 95% CI = 0.83–0.86, *ω* = 0.85) to generate a measure of moral self-relevance. Thus, the value of 0 indicates equal importance, positive scores reflect greater endorsement of moral traits for the self, and negative scores reflect a greater endorsement of non-moral traits (Barriga et al., 2001).

#### Individual moral disengagement

Moral disengagement was measured at T1 with a 22-item scale that has been extensively used with Italian adolescents (e.g., Caravita et al., 2019; Gini et al., 2015a, 2015b, 2020; Pozzoli et al., 2012). The scale has been initially developed and validated for Italian children and adolescents (Caprara et al., 1995) and its unidimensionality has been repeatedly confirmed (e.g., Pozzoli et al., 2016). Its validity has been replicated in various countries, such as China (Wang & Yang, 2010), Spain (Rubio-Garay et al., 2017), and the United States (Pelton et al., 2004). Examples of items are the following: “Some kids deserve to be treated like animals,” “Kids cannot be blamed for misbehaving if their friends pressured them to do it.” Participants rated their agreement with each statement on a 5-point scale, from 1 (*strongly disagree*) to 5 (*strongly agree*) and their responses were averaged to form a score of moral disengagement (*α* = 0.87; 95% CI = 0.87–0.88, *ω* = 0.87).

#### Collective moral disengagement

Collective moral disengagement was assessed at T1 with a 17-item scale designed and validated with Italian adolescents (Gini, Pozzoli, & Bussey, 2014). Each item describes moral disengagement mechanisms (e.g., “it is alright to beat someone who bad mouths your family”, “if kids fight and misbehave in school it is their teacher’s fault,” “children do not mind being teased because it shows interest in them,” “kids who get mistreated usually do things that deserve it”) that can be shared by classroom members. For each item students were asked “In your classroom, how many students think that… [item]” on the following 5-point scale: “None,” “About a quarter (25%),” “About a half (50%),” “About three quarters (75%),” and “Everyone.” The validation study demonstrated good factorial and criterion validity of the scale’s scores (Gini, Pozzoli, & Bussey, 2014). Further support for its validity has been found in other countries, such as Australia (Allison & Bussey, 2017), Czech Republic (Kollerová et al., 2018), and Turkey (Çapan & Bakioglu, 2016).

Following the original procedure, for each participant item scores were averaged to form a perceived collective moral disengagement score (*α* = 0.87; 95% CI = 0.86–0.88, *ω* = 0.87), to be used at the individual level. This score represents students’ perception of the degree to which classmates share moral disengagement mechanisms. The aggregate score of this scale at the class-level—that is, the average score of all classroom members—provides a measure of classroom collective moral disengagement.

### Analysis

First, longitudinal confirmatory factor analyses were conducted on the aggression scores at both waves to check for longitudinal invariance. A two-factor model (reactive and proactive aggression) with 12 items for each factor was tested, with each factor allowed to correlate at the two time points. Then, multivariate multilevel modeling was performed in Mplus 8.3 (Muthén & Muthén, 2017) with the full information maximum likelihood (FIML) estimator. The two simultaneous dependent variables were reactive and proactive aggression in spring (T2). These took into account the intercorrelation between the reactive and proactive aggressive behavior, while testing the relative strength of each of the respective predictors. At level 1, sex (0 = males, 1 = females), age, and fall levels of reactive and proactive aggression were entered as control variables. Moral identity, individual moral disengagement, and perceived collective moral disengagement were continuous predictors (all measured at T1). To enhance interpretability, individual level variables, except sex, were group-mean centered. In addition to main effects, the two-way interactions between the three moral predictors were entered. At level 2, class-level aggression scores were entered to control for the stability of the respective aggression score (i.e., class-level reactive aggression at T1 for T2 reactive aggression and class-level proactive aggression at T1 for T2 proactive aggression) and classroom collective moral disengagement was entered as key contextual predictor. Variables at the class-level were grand-mean centered.

Finally, it was examined whether there was between-classroom variability in the associations between individual predictors and T2 aggression (i.e., random slopes). In case a significant random slope emerged, cross-level interaction would be tested to check if a level-2 variable could explain the slope variability.

## Results

### Missing Data

There were minimal missing data due to non-response by a small number of students. Specifically, there were 3 missing scores (0.2%) for moral identity, 6 (0.5%) for perceived collective moral disengagement, and 11 for (0.8%) moral disengagement. Sex and age variables did not include any missing scores. To handle missing data, full information maximum likelihood (FIML) estimation (Enders & Bandalos, 2001) in Mplus was used, so that all available information was used in the model estimation.

### Longitudinal Invariance of Reactive and Proactive Aggression

As preliminary analyses, longitudinal confirmatory factor analyses were performed on the reactive and proactive aggression scores at T1 and T2 (Millsap & Cham, 2012). To evaluate whether the assumption of invariance is acceptable, change in value of fit indices (i.e., ΔCFI, ΔRMSEA, ΔSRMR) was considered. Negligible change, that is, a ΔCFI smaller than 0.01 and a change smaller than 0.015 in RMSEA and SRMR, was considered indicative of invariance (e.g., Chen, 2007; Cheung & Rensvold, 2002). First, configural invariance was tested by running an unconstrained model, that is, all parameters were freely estimated in the two waves. This analysis showed an acceptable fit: *χ*^2^ = 7857.4, CFI = 0.931, RMSEA = 0.068, SRMR = 0.071. Then, two subsequent analyses in which parameters were progressively constrained to be equal across waves confirmed metric invariance (ΔCFI = −0.011; ΔRMSEA = 0.006; ΔSRMR = −0.001), and scalar invariance (ΔCFI = −0.003; ΔRMSEA = 0.005; ΔSRMR = 0.000). Full results are reported in Table 1.

### Descriptive Statistics and Correlations

Tables 2 and 3 display descriptive statistics and bivariate correlations at the individual-level and the class-level, respectively. As expected, both types of aggressive behavior showed significant stability across the school year, both at the individual and the class-level. Moreover, moral identity was negatively associated with both types of aggression, which conversely were positively correlated with individual moral disengagement and perceived collective moral disengagement. At the class-level, classroom collective moral disengagement at T1 was positively correlated with class-level reactive and proactive aggression at both time points.

**Table 2:** Means, standard deviations and correlations among variables at the individual level

	*M*	SD	1.	2.	3.	4.	5.	6.
1. Reactive aggression T1	2.03	0.86	-					
2. Proactive aggression T1	1.45	0.76	0.74	-				
3. Reactive aggression T2	2.04	0.86	0.57	0.42	-			
4. Proactive aggression T2	1.49	0.83	0.41	0.46	0.76	-		
5. Moral identity T1	0.39	0.68	−0.33	−0.27	−0.31	−0.28	-	
6. Moral disengagement (MD) T1	2.04	0.60	0.58	0.47	0.46	0.36	−0.32	-
7. Perceived collective MD T1	1.98	0.60	0.43	0.40	0.35	0.32	−0.27	0.56

All *ps* < 0.001

**Table 3:** Means, standard deviations and correlations among variables at the class level

	*M*	*SD*	1.	2.	3.	4.
1. Reactive aggression T1	2.03	0.29	-			
2. Proactive aggression T1	1.46	0.28	0.78	-		
3. Reactive aggression T2	2.04	0.31	0.64	0.49	-	
4. Proactive aggression T2	1.49	0.35	0.50	0.48	0.88	-
5. Collective moral disengagement T1	1.98	0.29	0.59	0.56	0.63	0.55

All *ps* < 0.001

### Multilevel Analyses

Regarding the main analysis of the current study, first an empty random intercept multilevel model (i.e., unconditional model) was estimated to calculate how much variance of T2 reactive and proactive aggression existed at the individual and at the class-level. Within- and between-level variance estimates were 0.685 and 0.047 for reactive aggression, and 0.591 and 0.078 for proactive aggression. The intraclass correlation coefficients indicated that 6.4% of the variation of reactive aggression and 11.6% of the variation of proactive aggression were due to differences between classes. Therefore, the use of multilevel modeling with predictors at both the individual and class-level was justified.

In the multivariate analysis, reactive and proactive aggression at T2 were positively correlated (*r* = 0.31, *p* < 0.001). Results of the full multilevel model are summarized in Table 4. The model explained 34% of variance for T2 reactive aggression and 27% of variance for T2 proactive aggression at the individual level, and 61% and 36% of class-level variance for reactive and proactive aggression, respectively. For the sake of simplicity, the findings for reactive aggression and proactive aggression are reported separately. For each type of aggression, the results are reporting following the order of the study hypotheses.

**Table 4:** Multivariate multilevel modeling predicting T2 reactive and proactive aggression

	Reactive Aggression (T2)				Proactive Aggression (T2)			
	b	*SE*	*z*	*p*	b	*SE*	*z*	*p*
*Individual Level (T1)*								
Age	0.080	0.07	1.19	0.234	0.117	0.07	1.64	0.102
Gender (0 = M, 1 = F)	−0.186	0.05	−3.79	<0.001	−0.278	0.06	−4.89	<0.001
Reactive aggression	0.461	0.05	9.22	<0.001	0.030	0.05	0.64	0.523
Proactive aggression	−0.055	0.06	−0.98	0.327	0.342	0.05	6.35	<0.001
Moral identity	−0.094	0.04	−2.54	0.011	−0.113	0.04	−3.20	0.001
Individual moral disengagement (MD)	0.152	0.06	2.42	0.015	0.043	0.07	0.63	0.526
Perceived collective moral disengagement	0.060	0.05	1.27	0.204	0.100	0.05	2.15	0.031
Individual MD * moral identity	−0.025	0.06	−0.41	0.680	−0.154	0.07	−2.16	0.031
Collective MD * moral identity	0.007	0.07	0.11	0.916	0.107	0.06	1.77	0.077
Individual MD * collective MD	−0.010	0.06	−0.17	0.868	0.007	0.08	0.09	0.926
*Class Level*								
Class-level aggression	0.408	0.08	5.09	<0.001	0.338	0.09	3.67	<0.001
Classroom collective MD	0.304	0.12	2.54	0.011	0.328	0.13	2.58	0.010
*R*^*2*^_individual level_	0.34				0.27			
*R*^*2*^_class level_	0.61				0.36			

*b* indicates unstandardized estimates from multilevel modeling

#### Reactive aggression

At the individual level, T1 reactive aggression was significantly associated with T2 reactive aggression (*b* = 0.461, SE = 0.05, *p* < 0.001), showing that aggressive behavior was quite stable. Conversely, T2 reactive aggression was not significantly predicted by T1 proactive aggression, thus confirming the validity of distinguishing between the two forms of aggression. Regarding the first hypothesis of significant prospective effects of individual moral factors on aggressive behavior, students who reported lower moral identity (*b* = −0.094, SE = 0.04, *p* = 0.011) and higher moral disengagement at T1 (*b* = 0.152, SE = 0.06, *p* = 0.015) reported higher levels of reactive aggression at T2 (controlling for baseline reactive aggression). Of the control variables, only sex was a significant predictor (*b* = 0.186, SE = 0.05, *p* < 0.001), indicating that males were more likely than females to report higher reactive aggression at T2. Contrary to the hypothesis, none of the two-way interactions was significant for reactive aggression.

The last hypothesis concerned the class-level effect of collective moral disengagement on aggressive behavior. After accounting for the stability of class aggression, higher levels of reactive aggression in spring were found in classrooms with higher collective moral disengagement in fall (*b* = 0.304, SE = 0.12, *p* = 0.011).

#### Proactive Aggression

**Fig. 1:**
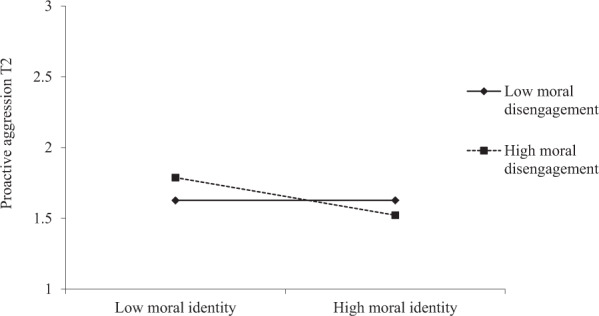
Moral Identity x Moral Disengagement Interaction

As far as proactive aggression is concerned, controlling for its stability over the school year and for the positive effect of sex, the analysis at the individual level yielded two significant main effects and one interaction. Regarding the first hypothesis, students who reported lower moral identity at T1 reported more proactive aggression at T2 (*b* = −0.113, *SE* = 0.04, *p* = 0.001). This main effect was qualified by a significant interaction between moral identity and individual moral disengagement. Simple slope analysis revealed that the negative association between moral identity and T2 proactive aggression was apparent at high (+1 *SD*) levels of moral disengagement (*b* = −0.204, *p* = 0.001) but not at low levels (-1*SD*) of moral disengagement (*b* = 0.000, *p* = 0.99). As shown in Fig. 1, proactive aggression at T2 was higher when students had lower moral identity and higher moral disengagement. There was also a significant main effect for perceived collective moral disengagement: Proactive aggression was significantly predicted by higher perceived collective moral disengagement (*b* = 0.100, *SE* = 0.05, *p* = 0.031).

Regarding the last hypothesis, at the class level, similar to the findings for reactive aggression, higher proactive aggression at the end of the school year was predicted by higher classroom collective moral disengagement (*b* = 0.328, *SE* = 0.13, *p* = 0.010).

As a final step, it was tested whether the slopes of the associations between the three moral predictors and T2 aggressive behavior varied across classrooms. No significant cross-level interactions emerged for either reactive aggression or proactive aggression (Results are reported in Appendix 2 – Supplementary material). In the interest of parsimony, cross-level interactions were not included in the final model.

## Discussion

Although psychological research has analyzed the risk factors for adolescent aggressive behavior within the moral domain, there is still a lack of integration of different moral correlates into a single model, as well as very limited analysis of class-level moral dimensions, especially in a longitudinal framework. To contribute to filling these gaps, in the current study moral identity and moral disengagement were both considered as potential longitudinal predictors (in opposite directions) of aggressive behavior. Moreover, another novelty of this study was the analysis of moral disengagement both as individual trait-like characteristic, describing the personal tendency to morally disengage, and as class-level collective characteristic, referring to the degree to which classmates agree with morally disengaged judgments. Finally, two types of aggressive behavior, namely reactive and proactive aggression, were included adding to the current literature that has focused on either general aggression or specific subtypes of aggressive behavior.

### Sex Differences and Stability of Aggressive Behavior

First, consistent sex differences emerged with males reporting more frequent T2 aggressive behavior than females. This effect was similar for both reactive and proactive aggression. Moreover, as one would expect and consistent with previous findings (McAuliffe et al., 2006), reactive and proactive aggression showed moderate stability over 6 months, both at the individual and the class-level. This indicates that not only individual students who are more aggressive in the fall tend to report more aggressive behavior at the end of the school year, but also that school classes that show, on average, more frequent aggressive behavior in the fall tend to remain high on aggression rates compared to other class-groups. Taking this stability into account, as well as the intercorrelation between the two forms of aggression, the findings showed that both individual and class moral dimension can prospectively predict adolescent aggression.

### Moral Identity and Moral Disengagement

As hypothesized, and consistent with theories of self and social-cognitive theory of morality (Hardy et al., 2020), moral identity was confirmed as protective factor for both reactive and proactive aggression. Indeed, students who scored higher on moral identity in the fall reported lower levels of both reactive and proactive aggressive behavior in the spring, compared to their classmates. In other words, valuing moral characteristics as central to the self may motivate adolescents to avoid adopting immoral actions, including aggression, to preserve the coherence of their self-image (Stets & Carter, 2011). This finding confirms and adds to previous cross-sectional studies that have shown that high centrality of moral characteristics for defining one’s self is negatively associated with the enactment of aggressive (Pozzoli et al., 2016) and antisocial behavior (Hertz & Krettenauer, 2016).

Interestingly, a difference between the two forms of aggression emerged, in that, while the main effect of moral identity was significant for both types of aggressive behavior, for proactive aggression it was also qualified by a significant interaction between moral identity and individual moral disengagement. This result indicated that moral identity was negatively associated with proactive aggression only when adolescents also reported high levels of moral disengagement. In particular, as shown in the graphical depiction of the interaction, the highest levels of proactive aggression were found when students had a combination of low moral identity and high moral disengagement. This may be associated with the nature of proactive aggression, which is an unprovoked use of coercion to attain a goal, whether that goal involves material gain or social dominance (Hubbard et al., 2010). A combination of low moral identity with an individual tendency to morally disengage seems to exacerbate the individual’s proneness to instrumentally use aggression to gain social advantages or dominate others. Because youth who use instrumental aggression may be more likely to morally disengage if they foresee emotional or material gains resulting from the transgression (Arsenio et al., 2009; Gasser & Keller, 2009), in future studies it would be interesting to replicate the current findings by also testing the role of adolescents’ expectations for their use of aggressive behavior. Moreover, this interaction effect supports the protective role moral identity can play in the context of peer aggression. Specifically, youth who reported high levels of moral disengagement displayed less proactive aggression 6 months later if they reported high moral identification. This is consistent with the tenets of social cognitive theory (Bandura, 2016) according to which the effects of moral disengagement can be lessened by an individual’s personal factors, including their moral identity.

### Collective Moral Disengagement

Another important novelty of this study was the analysis of the role of collective moral disengagement in a longitudinal design. As discussed in the introduction, collective moral disengagement can be conceptualized and measured both at the individual and the group level (Gini et al., 2015b). As an individual dimension, perceived collective moral disengagement refers to individuals’ beliefs about the extent to which members of their group use moral disengagement mechanisms. In this study, this perception was positively associated with higher proactive aggression 6 months later in a model that accounted for the role of moral identity and individual moral disengagement. Even though the zero-order correlation between perceived collective moral disengagement and reactive aggression was significant, this association became non-significant in the final model that partialled out the effects of the other moral variables. As hypothesized, therefore, a difference between proactive and reactive aggression emerged, indicating that group processes— in terms of the individual perception of how much moral disengagement was normative in the class group— played a stronger role in proactive aggression than in reactive aggression. This suggests that, beyond the effect of students’ own tendency to morally disengage, adolescents who perceive that moral disengagement mechanisms are shared among their classmates may be more prone to accept aggressive behavior as “normal” and acceptable, and to instrumentally use it as means to handle peer relationships within the group. This finding therefore confirms what has been found in other longitudinal studies about the role of peers’ moral disengagement in influencing adolescents’ proactive aggression (Sijstema et al., 2014).

At the class-level, collective moral disengagement captures a collective property of a class-group reflecting the degree to which moral disengagement mechanisms are shared by classmates. Findings from this sample of adolescents confirm and expand previous cross-sectional studies by showing that, even after controlling for its stability across 6 months, aggressive behavior was more likely in school classes characterized by higher levels of collective moral disengagement (Gini et al., 2015b). Social psychological research has shown that diffusion of responsibility, victim blame, dehumanization, and similar processes can lead members of societies or small social groups to enact, overtly support, or silently approve harmful behavior (e.g., Haslam, 2006). This study shows that these group mechanisms may also operate to influence aggressive behavior in school classes, whether it is a reactive aggression or an instrumental use of coercion. Thus, in a classroom, it may be that the more adolescents aggress against others as a group, the less they feel responsible for their harmful behavior. Because of the relevance of group mechanisms for intervention programs, future studies should seek to confirm and replicate the current findings in other samples and different age groups. Moreover, it would be interesting to test for the longitudinal role of collective moral disengagement for other types of negative behavior, as well as for positive, prosocial behavior, such as defending and helping victimized classmates.

### Limitations and Implications for Interventions

One limitation of the current study is the relative short interval between the first and the second data collection. Even though the adoption of a longitudinal design is a strength of the current study, due to the abundance of cross-sectional studies in this field, the conclusion that can be drawn from our analyses are limited to the period of about six months. The choice of this time interval was based on the necessity of capturing the group dynamics within a single school-year. Further investigations that extend to more than one school-year and possibly follow adolescents for more years are warranted. Moreover, we did not collect data in three or more waves. This precluded any analysis of the trajectories of aggressive behavior and more analytic tests of how the associations between the predictors and aggression change over a longer time period.

Another limitation is the extent to which the current findings on moral identity can be generalized across cultures, or particular subgroups of youth. For example, research on moral identity has showed that moral identity predicts moral action more often in Western cultures than in non-Western cultures (Jia & Krettenauer, 2017). Although the general concept of moral identity may exist in all cultures, it may function in different ways and may be somewhat context-dependent tied to varying social and cultural obligations. It is not necessarily the case, therefore, that these findings from the Italian school context can be generalized to other cultural contexts, especially non-Western contexts. More research adopting a cross-cultural lens is warranted in this field.

Finally, another possible limitation of this study is the use of only self-reports. Reliance on self-report data may lead to problems, such as social desirability bias (Krumpal, 2013); however it should also be noted that it is often the best (or only) choice available to researchers. The research questions of the current study mainly related to beliefs and internal processes of adolescents and the individual is the only one who can report on such variables. Also regarding aggressive behavior, even though peer nominations or peer ratings could be used, it is not necessarily true that adolescents are good informants of other peers’ aggressive behavior; in some circumstances, the individual is the only one who knows certain aspects of their behavior, for example, when aggression consists of more covert behavior. Nonetheless, it would be important to confirm the findings of the present work in longitudinal studies that employ multiple sources of information about adolescents’ aggressive behavior, such as peers (Branson & Cornell, 2009).

These limitations notwithstanding, the current findings contribute significant knowledge to the literature on the role of moral identity and moral disengagement in aggressive behavior. The findings further suggest the need to include a moral component in school-based anti-aggression and anti-bullying interventions. This could be achieved by adding specific modules to already existing evidence-based interventions, such as the Olweus Bullying Prevention Program (Olweus & Limber, 2010) or the Positive Behavioral Interventions and Supports (Bradshaw, 2013). These comprehensive intervention programs already include activities to be conducted within each classroom or with small groups of schoolmates and they could be easily adapted to include moral modules, where students are provided with clear messages about the moral standard that explicitly states the unacceptability of aggression and bullying under all circumstances. It is also essential that a whole-school approach involving each of the classes in the school is used as our research shows that not only was individual level moral disengagement associated with aggression, but collective moral disengagement was longitudinally predictive, over six months, of reactive and proactive aggression. It is therefore necessary to message the entire peer group, not just the aggressors, about the immorality of aggression. These messages could be enhanced by using cognitive behavior techniques that challenge moral disengagement mechanisms that justify aggressive behavior. For example, the methods used by Aly et al. (2014) to counter the radicalizing influences of extreme violence beliefs could be adapted to counter aggressive behavior coupled with the promotion of positive moral values to enhance moral identity. Students in each class, for example, could be encouraged to adopt the different components of moral identity and each month a student who exhibits such positive behaviors is named on the school’s honor notice board. Considering the protective role of moral identity that emerged in this study, schools should consider developing a culture favoring the development of adolescents’ moral identity, valuing the importance on being “a good person” and elevating it to a central component of self-identity. Further guidance for implementing methods to promote moral identity are provided by Narvaez and Bock’s (2014) ethical education program, which include (a) establishing a caring relationship with each student, (b) establishing a climate supportive of achievement and ethical character, (c) teaching ethical skills in terms of ethical sensitivity, ethical judgment, ethical focus and ethical action, and (d) fostering student self-authorship and self-regulation. In sum, our study highlights the need for future school-based anti-aggression and anti-bullying interventions to include a moral component at both the individual and class levels.

## Conclusion

Past research has showed that morality is central to the understanding of the development of adolescent aggressive behavior. However, there is a dearth of longitudinal studies that test the main and interactive effects of multiple moral factors simultaneously, and that operationalize these factors both at the individual and the class-level. The current study used a longitudinal, multilevel approach to analyze the associations of moral identity, individual moral disengagement, and collective moral disengagement with reactive and proactive aggression over 6 months. As expected, reactive and proactive aggression were quite stable over this short period of time. At the individual level, the results confirmed the protective role of high moral identity against the enactment of both forms of aggression. Individual moral disengagement was also confirmed as potential risk factor for reactive aggression. For proactive aggression, a significant interaction effect indicated that the negative effect of moral disengagement was attenuated by moral identity. Finally, a novel contribution of this study was to confirm that high collective moral disengagement, as class-level property, is also a risk factor for more frequent aggressive behavior. By understanding the relative strength of different moral factors for each form of aggression and by highlighting that moral mechanisms can also play a role at the group level, researchers can better inform prevention and intervention efforts with adolescents. In particular, this study underscores the importance of explicitly including a moral component in school-based anti-aggression interventions.

## Supplementary Information

Supplementary Materials
