# Supplementary material for: Longitudinal Links of Individual and Collective Morality with Adolescents’ Peer Aggression
Source: J Youth Adolesc. 2021 Oct 18;51(3):524–39. doi: 10.1007/s10964-021-01518-9 (PMC8881436; doi:10.1007/s10964-021-01518-9)
Supplement: Supplementary file 1 — Supplementary Materials [file 10964_2021_1518_MOESM1_ESM.docx]

**Supplementary Materials**

**Longitudinal Links of Individual and Collective Morality with Adolescents’ Peer Aggression**

*Journal of Youth and Adolescence*

Gianluca Gini^1^, Robert Thornberg^2^, Kay Bussey^3^, Federica Angelini^1^, & Tiziana Pozzoli^1^

*^1^University of Padova, Italy*

*^2^Linköping University, Sweden*

*^3^Macquarie University, Australia*

Contact corresponding author: [gianluca.gini@unipd.it](mailto:gianluca.gini@unipd.it)

**Appendix 1.**

Results of attrition analysis: comparison between students who participated in both waves and those who did not.

|  | Participants  at T1 and T2 | | Participants  missing at T2 | | *t* | *p* |
| --- | --- | --- | --- | --- | --- | --- |
|  | *M* | *SD* | *M* | *SD* |  |  |
| Age | 13.60 | 1.12 | 14.18 | 1.05 | 5.44 | <.001 |
| Reactive aggression | 1.03 | .86 | 2.09 | .83 | -.745 | .456 |
| Proactive aggression | 1.45 | .76 | 1.47 | .66 | -.231 | .817 |
| Moral identity | .39 | .68 | .38 | .71 | .120 | .904 |
| Individual moral disengagement | 2.04 | .60 | 2.08 | .61 | -.689 | .491 |
| Perceived collective moral disengagement | 1.98 | .60 | 2.00 | .60 | -.328 | .743 |

**Appendix 2.**

Results of analyses on cross-level interactions. None of the interaction was statistically significant and they were dropped from the final model.

|  | Reactive Aggression (T2) | | | |  | Proactive Aggression (T2) | | | | |
| --- | --- | --- | --- | --- | --- | --- | --- | --- | --- | --- |
| *Random slope* | b | *SE* | *z* | *p* |  |  | b | *SE* | *z* | *p* |
| Moral identity | 0.010 | 0.007 | 1.44 | .149 |  |  | 0.004 | 0.006 | 0.67 | .506 |
| Individual moral disengagement | 0.005 | 0.009 | 0.55 | .579 |  |  | 0.019 | 0.014 | 1.32 | .186 |
| Perceived collective moral disengagement | 0.019 | 0.013 | 1.47 | .142 |  |  | 0.015 | 0.012 | 1.24 | .215 |

Note. The estimate b is the variance of the slope, set as random.
